# Deep Brain Stimulation for Obsessive-Compulsive Disorder: Real World Experience Post-FDA-Humanitarian Use Device Approval

**DOI:** 10.3389/fpsyt.2021.568932

**Published:** 2021-03-24

**Authors:** Lora Kahn, Brianne Sutton, Helena R. Winston, Aviva Abosch, John A. Thompson, Rachel A. Davis

**Affiliations:** ^1^Department of Neurosurgery, Ochsner Health, Tulane University-Ochsner Health Neurosurgery Program, New Orleans, LA, United States; ^2^Department of Psychiatry, University of Colorado, Anschutz Medical Campus, Aurora, CO, United States; ^3^Department of Neurosurgery, University of Nebraska Medical Center, Omaha, NE, United States; ^4^Department of Neurosurgery, University of Colorado, Anschutz Medical Campus, Aurora, CO, United States; ^5^Department of Neurology, University of Colorado, Anschutz Medical Campus, Aurora, CO, United States

**Keywords:** psychiatric DBS, co-morbidity, deep brain stimulation, obsessive-compulsive disorder, DBS programming

## Abstract

**Background:** While case series have established the efficacy of deep brain stimulation (DBS) in treating obsessive-compulsive disorder (OCD), it has been our experience that few OCD patients present without comorbidities that affect outcomes associated with DBS treatment. Here we present our experience with DBS therapy for OCD in patients who all have comorbid disease, together with the results of our programming strategies.

**Methods:** For this case series, we assessed five patients who underwent ventral capsule/ventral striatum (VC/VS) DBS for OCD between 2015 and 2019 at the University of Colorado Hospital. Every patient in this cohort exhibited comorbidities, including substance use disorders, eating disorder, tic disorder, and autism spectrum disorder. We conducted an IRB-approved, retrospective study of programming modifications and treatment response over the course of DBS therapy.

**Results:** In addition to patients' subjective reports of improvement, we observed significant improvement in the Yale-Brown Obsessive-Compulsive Scale (44%), the Montgomery-Asberg Depression Rating Scale (53%), the Quality of Life Enjoyment and Satisfaction Questionnaire (27%), and the Hamilton Anxiety Rating scales (34.9%) following DBS. With respect to co-morbid disease, there was a significant improvement in a patient with tic disorder's Total Tic Severity Score (TTSS) (*p* = 0.005).

**Conclusions:** DBS remains an efficacious tool for the treatment of OCD, even in patients with significant comorbidities in whom DBS has not previously been investigated. Efficacious treatment results not only from the accurate placement of the electrodes by the surgeon but also from programming by the psychiatrist.

## Introduction

Obsessive-compulsive disorder (OCD) is a debilitating disorder characterized by obsessions and compulsions that afflicts ~1.2% of people in the United States and between 1.1 and 1.8% worldwide (1). Obsessions are unwanted thoughts, urges, or images that cause distress. Compulsions are repetitive behaviors or mental “acts” (such as counting) that are performed to assuage distress or to prevent a feared event from happening. Many but not all compulsions make sense cognitively but consume far more time than they would for someone without OCD. For example, fear of contamination might lead to excessive handwashing or fear of burning down the house might lead to excessive checking of the stove. While there are different severities of OCD, some people suffer extreme impairment to the degree that they are unable to maintain regular employment or enjoy everyday activities (2). There are five general subsets of symptoms within OCD, including contamination obsessions with washing/cleaning compulsions; harm obsessions with checking compulsions, obsessions without visible compulsions; symmetry obsessions with ordering, arranging, and counting compulsions, and hoarding (3).

Treatment generally includes cognitive-behavior therapy (CBT) with exposure and response prevention (EX/RP) alone or a combination of EX/RP and medications such as selective serotonin reuptake inhibitors (SSRIs), the tricyclic antidepressant clomipramine, and/or antipsychotics (4, 5). Despite maximal treatment, usually combining EX/RP with serotonergic and other augmenting agents, it is seldom that patients with OCD are able to achieve full remission, which is defined as a subclinical score of ≤ 7 on the Yale-Brown Obsessive Compulsive Score (Y-BOCS). Approximately 10% remain severely incapacitated despite receiving EX/RP coupled with therapeutic medication regimens (6). For these refractory patients, treatment options are extremely limited.

Deep brain stimulation (DBS) involves a technique by which stimulating electrodes are placed in the deep nuclei of the brain, usually the ventral capsule/ventral striatum. The mechanism of DBS in OCD has not been fully elucidated but is thought to modify aberrant circuitry, including the cortico-striato-thalamic-cortical (CSTC) circuit. Applied initially to intractable pain, DBS is most commonly employed in movement disorders such as Parkinson's disease but has been used for the treatment of OCD predicated on the understanding of the CSTC circuit's involvement in this disorder (7). The idea of applying DBS to OCD grew out of observations that lesional procedures such as anterior capsulotomy, utilized for the treatment of OCD since the 1950s, are about 50–60% effective in treating refractory patients with the disorder (8–10). However, whereas lesional procedures create enduring changes in the brain by permanently destroying tissue and irreversibly interrupting circuits, DBS is a reversible and titratable form of neuromodulation.

The first case of DBS for refractory OCD was performed in 1999 in the anterior limb of the internal capsule (ALIC; the same target as in anterior capsulotomy) before being further refined to the ventral capsule/ventral striatum (VC/VS). Both the AC and VC/VS participate in the same CSTC circuit (10, 11). In fact, stimulating different targets within the CSTC circuit has been shown to have similar efficacy (12). VC/VS is the most commonly reported target in the literature, followed by the nucleus accumbens (NAc) and then others (13). Bilateral targeting is performed in DBS surgery as the 2014 evidenced-based guidelines reported that there are insufficient data to support unilateral targeting (14). DBS received a Humanitarian Use Device (HUD) designation in 2009 under a Humanitarian Device Exemption (HDE), meaning that it can be used to treat “severe to extreme” refractory cases of OCD. An HDE is granted for Humanitarian Use Devices (HUDs) that have been found to be safe, have probable benefit, and are intended to be used in <8,000 patients per year. HDEs are designed to bring hope to those suffering severely who cannot wait for extensive large-scale trials that would be required to demonstrate the effectiveness and may never be feasible (15).

While previous studies have established that DBS for OCD is likely to be a beneficial treatment for refractory severely impaired patients, these studies have largely ignored how DBS impacts (or does not affect) the other psychiatric diseases that are so frequently comorbid with OCD (12). OCD rarely occurs in isolation. For example, according to the DSM5, 76% of patients with OCD also have a lifetime diagnosis of an anxiety disorder such as panic disorder, generalized anxiety disorder, or social anxiety disorder; 41% have a lifetime diagnosis of major depressive disorder (MDD); 22% have a lifetime diagnosis of a bipolar spectrum or depressive disorder other than MDD; and 30% have a lifetime tic disorder. The DSM5 also states that rates of OCD are elevated in those with eating disorders and schizophrenia-spectrum disorders. Here we discuss our DBS treatment of refractory OCD patients who have such comorbid disease and our experiences with programming for OCD while managing multiple symptoms of these other illnesses and minimizing side effects.

## Methods

Between 2015 and 2019, five patients were implanted bilaterally with 4-contact electrodes (Model 3391, Medtronic, Minneapolis, MN) targeting the VC/VS at our institution by a single surgeon (AA) after approval by an interdisciplinary ethics conference as advised in the literature (16). Three cases were done awake with microelectrode recording and intraoperative testing by a single psychiatrist (RD), and the remaining two patients elected for an asleep protocol using an MRI-guided direct targeting technique. Consensus coordinates were utilized for initial targeting; however, the targets were ultimately refined directly based on each patient's individual images. The indirect targeting anatomic coordinates used were 7–10 mm lateral to the midline on the X axis, 0–5 mm anterior to the anterior commissure (AC) in the Y axis, and 1–5 mm inferior to the inferior border of the AC in the Z axis. In all cases, the target was advanced by 3 mm (the depth of one contact) after the identification of the direct target to allow for the space between contacts 0 and 1 to rest at the junction of the anterior limb of the internal capsule (ALIC) with the anterior limb of the AC. All patients returned at least 1 week after cranial lead implantation for placement of bilateral extension cables and pulse generators. There were no associated surgical complications. Rating scales were performed by a single psychiatrist (RD) who was also the primary programmer; when multiple scales were available from pre-operative assessment, they were averaged for the sake of our analysis. We have from 1 to 4 years of follow-up for each patient. Patients provided informed, written consent for this retrospective case report and reviewed the material described in this report; in addition, all efforts have been made to preserve anonymity.

### Calculation of Charge Density

For monopolar configurations, charge density was calculated with the standard approach:

Charge density = (current ^*^ PW)/surface area. For bipolar configuration, charge density was calculated by dividing the current at the cathode and anode.

### Measurement of Distance Between the Active Contact(s) and the Anterior Commissure (AC) – Anterior Limb of the Internal Capsule (ALIC) Junction

For each patient, using pre-operative MRI, expert identification of the AC-ALIC junction was localized to the axial plane at the optimal level for the AC. Following co-registration of the pre-operative MRI with the post-operative CT (in cases 2–5) or post-operative MRI (case 1), the ventral-most point of the lead artifact was localized. Next, for each patient, the final follow-up active contacts were used to estimate the location along the lead artifact for localizing the active contact in the CT space. For a monopolar setting, the midpoint of the contact was used; for bipolar or double monopolar settings, the midpoint between the contacts was used as the active contact location. Finally, the distance in mm between the active contact location along the electrode artifact and the AC-ALIC junction was measured.

### Methodology for Programming

A single psychiatrist (RD) performed initial and ongoing programming for all 5 patients. Initial programming took place over three consecutive days, then weekly for several weeks, followed by every other week for about 6 weeks, then monthly with spacing to every 3 months once ideal settings were selected. The programming algorithm described by Widge et al. (17) was followed on the first 3 days, with adjustments to the algorithm as needed based on patient response (e.g., titrating in smaller increments for patient comfort or fine-tuning, not increasing to 6 V if the response was obvious at 4 V). Selection of parameters was based on a reduction in anxiety, an increase in energy, improvement in mood, the patients' subjective experience, and the programmer's observations of the patient's engagement and affect (18). On day one, the psychiatrist performed a monopolar survey at contacts 0, 1, 2, and 3 at amplitudes of 2, 4, and 6 V with frequency of 135 and pulse width of 90 microseconds. This was repeated at a pulse width of 150 microseconds and was done separately for each hemisphere. On day two, the psychiatrist performed a bipolar survey (with contact 3 as the anode) at each contact using a frequency of 135 and the pulse width value that yielded the best response during the monopolar survey. Widge et al., suggest using (0–, 1–, 3+) and (1–, 2–, 3+) (17). The psychiatrist in this report used (0–, 3+), (1–, 3+), (2–, 3+) or the combination of 2 cathodes as suggested by Widge et al. depending on patients' response during the monopolar survey (17). On day three, the psychiatrist selected the settings at which the patient had the best response and made minor adjustments as needed, such as increasing or decreasing amplitude, decreasing frequency (e.g., to target increased anxiety), or decreasing or increasing pulse width (e.g., if a patient had more improvement at 150 microseconds but also more adverse effects, an intermediate pulse width could be selected).

### Statistical Analysis for Diagnostic Rating Scales

For all five patients, the following scales were assessed before DBS surgery and at every programming session after implantation: the Yale-Brown Obsessive Compulsive Scale (Y-BOCs), the Montgomery-Asberg Depression Rating Scale (MADRS), the Hamilton Anxiety Rating Scale (HAM-A), and the Quality of Life Enjoyment and Satisfaction questionnaire (Q-LES-Q-SF), and the Young Mania Rating Scale (YMRS). Individual cases exhibited comorbidities that were assessed with relevant scales: Case 1, with a history of anorexia nervosa, assessed with Eating Disorder Examination 16.0 (19); Case 2, with a history of tic disorder, assessed with Yale Global Tic Severity Scale (YGTSS) (20); Case 4, had comorbid substance use which was assessed substance craving scales for cigarettes, marijuana and alcohol (21). Rating scale scores collected following DBS were compared to the pre-surgical baseline by computing the percent change. For each case, on each scale, the change from baseline was statistically assessed by a univariate paired *t*-test between the average pre-surgery and post-surgical assessment data. A Bonferroni correction was for multiple comparisons (per scale, the number of comparisons was equal to the number of cases).

### Case Vignettes

#### Case 1

A 32-year-old woman with a 24-year history of OCD and comorbid severe and enduring anorexia nervosa and severe major depressive disorder (MDD) presented with a Yale-Brown Obsessive-Compulsive Scale (YBOCS) rating of 36. Her obsessions included fear of bad things happening (of which one potential bad thing was weight gain), and her compulsions included repeating things a certain number of times, organizing and arranging, and reassurance seeking. Though she had previously worked briefly as a registered nurse, she had been institutionalized for much of her child and adult life. She had one previous suicide attempt in 2013, and she continued to experience persistent, passive suicidal thoughts. She had episodes of self-harm, including an incident where she fractured her hand 18 months prior to evaluation. She continued to engage in self-harm when distressed, including scratching and excoriating herself. She had failed numerous medications [8 adequate trials of serotonergic medications, 7 atypical antipsychotics, 2 first generation antipsychotics, 2 monoamine oxidase inhibitors (MAO-Is), 4 benzodiazepines, intranasal ketamine, and multiple augmenting agents] and electroconvulsive shock therapy. She elected to proceed with awake placement of bilateral VC/VS electrodes, and intraoperative exposure included the soft drink Coca-Cola, the candy Tootsie Pop, and the color red, to all of which she had an aversion.

#### Case 2

A 46-year-old man with a 25-year history of OCD together with autism-spectrum disorder, tic disorder, and MDD had failed 5 serotonergic medications including clomipramine at adequate dose and duration with appropriate augmenting strategies prior to presenting with a YBOCS of 39. His main obsession was that he was not seeing things correctly, and his compulsions included staring and checking. Despite doing well in advanced classes in high school, he was not able to finish higher education or maintain a job and thus elected to proceed with asleep direct targeting protocol placement of bilateral VC/VS electrodes. Details regarding Case 2 were previously published (22).

#### Case 3

The third patient was a 28-year-old man with a 19-year history of OCD together with attention deficit/hyperactivity disorder (ADHD), MDD, and a previous history of cannabis use disorder who failed multiple medications and augmenting strategies, including three trials of serotonergic medications at adequate dose and duration (one of which was clomipramine) and subsequently presented for treatment with a YBOCS of 32. His obsessions included disgust related to fast food, people who ate fast food, American cars, and anything/anyone from the East or the South. His compulsions including cleaning and washing. He had to withdraw from his graduate program but remained highly motivated to “be better.” He underwent staged, awake placement of bilateral VC/VS electrodes.

#### Case 4

A 48-year-old male had been diagnosed with OCD at age 24 by a priest because he presented compulsively to confession. At the time of presentation, he had a YBOCS of 32 after having failed 3 serotonergic medications at adequate dosage and duration and 2 antipsychotics. His obsessions included a fear of displeasing God, a fear of going to Hell, and a fear of his mother being in Hell. His compulsions included praying and moving in certain ways. He had comorbid MDD, insomnia, and issues with substance use [nicotine use disorder, daily cannabis use, and heavy alcohol use – as defined by the NIAAA (23)]. He was working in construction at the time of surgery. He underwent staged, awake placement of bilateral VC/VS electrodes.

#### Case 5

The most recently operated patient is a 42-year-old man with a 23-year history of OCD with comorbid MDD and social anxiety disorder who presented with a YBOCS of 36. His obsessions included a fear that inanimate objects were watching him play video games and that if he saw people moving or speaking, this would mean he wouldn't be able to move or speak in the future. He recognized the illogical nature of these thoughts and referred to them as “psychotic.” Though patient's obsessions were bizarre and irrational, he had good insight into this and did not meet criteria for a primary psychotic disorder. He had tried five different classes of medications, including 3 serotonergic medications including clomipramine at adequate dose and duration, benzodiazepines, antipsychotics, stimulants, and mood stabilizers. He had intravenous ketamine and underwent 40 sessions of deep transcranial magnetic stimulation (TMS) for OCD with limited effect. He had previously undergone a parathyroidectomy (pathology: normal) in attempt to ameliorate his symptoms; however, this did not result in the desired functional improvement. He subsequently elected to proceed with asleep-protocol bilateral placement of VC/VS electrodes.

### Programming

#### Patient 1

This patient agreed to remain in whatever level of care was necessary to maintain ideal body weight during the first year of DBS programming, which was ultimately residential treatment. Positive effect on mood and energy was partially maintained by turning down amplitude bilaterally at night. Currently, stimulation amplitude is set higher relative to the other patients in this cohort, and the authors postulate this is due to two factors: (1) severe, profound depression at baseline [highest score on MADRS of the 5 patients – 41.67 mean pre-operative score vs. 29.67 (#2), 28.5 (#3), 30 (#4), and 35.5 (#5)] and (2) less obvious response to stimulation led to continued titration.

#### Patient 2

Programming was complicated by this patient's autism spectrum disorder leading to difficulty describing his internal mood and anxiety states. He disliked any obvious changes so amplitude was increased very gradually, and frequency was lowered to 100 Hz.

#### Patient 3

The left electrode was pulled back post-operatively due to imaging showing it was abutting the internal carotid artery. There was still noted benefit during initial programming, but the patient felt the effect was less noticeable than the right. This patient experienced dramatic reduction in YBOCS and improvement in mood in the first week (YBOCS: 9 = 72% reduction; MADRS: 12 = 58% reduction; YMRS = 1) with R hemisphere: case +/0–/1–; 3.5 V; 135 Hz; 150 μs and L hemisphere: case +/0–; 4 V; 135 Hz; 150 μs. To this patient's dismay, these effects did not last, and his Y-BOCS increased back to 24 (27% reduction from baseline) and MADRS to 32 (12% increase from baseline) by the second week. He was quite disappointed for several months, hoping the psychiatrist would do something to bring back those feelings. His MADRS peaked at 37 (30% increase from baseline) with a Y-BOCS of 18 at 7 weeks post-stimulation. At this point, low-dose olanzapine (2.5 g) was added, leading to marked improvement in OCD and depression symptoms. MADRS declined to 11 at 14 weeks post-stimulation with a Y-BOCS of 16, then increased again to MADRS of 31 and Y-BOCS of 22 at 32 weeks post-stimulation after a month's trial of reduction in pulse width from 150 to 120 μs (reduced due to patient feeling jittery and agitated at amplitudes higher than 2 V on the right). Mood and OCD symptoms improved with increase back to 150 μs bilaterally, and he limited amplitude on the right to 2.6 V or less when in monopolar configuration. He has been on stable settings for the past 7 months and switches the right settings between monopolar (case+/1–; 2.4-2.6 V; 150 μs; 135 Hz) for sleep to bipolar (0+/1–; 5 V; 150 μs; 135 Hz) for work, school, or driving. He keeps the left at C+/2–; 4.0 V, 150 μs, 135 Hz.

#### Patient 4

This patient experienced transient improvement in OCD symptoms (29% reduction in Y-BOCS at 16 weeks) with relapse to 1 point higher than baseline at 3 weeks. He experienced marked dysphoria and irritability when pulse width was increased to 210 at 28 weeks post-stimulation. This resolved with temporary addition of olanzapine 5 mg (at 32 weeks) and decrease back to a pulse width of 150. At 71 weeks, patient's Y-BOCS had decreased to 25, and he described his remaining compulsions as reflexive and habit-like. The psychiatrist conceptualized his residual movement-related compulsions as “tourettic” (24), so haloperidol was added and titrated to 5 mg at bedtime. The patient experienced a marked reduction in Y-BOCS to 16 over the next 8 weeks without further change to DBS parameters.

#### Patient 5

This patient experienced early, marked improvement at low amplitude and pulse width. He began to experience hypomania with marked irritability at (R: case +/1–; 2.7 V; 90 μs; 135 Hz and L: C+/0–; 2.7 V; 90 μs; 135 Hz). Attempts to taper paroxetine (decrease from 80 to 60 mg/day) led to increase in intrusive thoughts. Patient did not tolerate trials of valproic acid and lithium. Irritability and hypomania remitted with change to bipolar settings at 21 weeks post-stimulation (R: 0–/3+; 4.5 V; 90 μs; 135 Hz and L: 0–/3+; 4.2 V; 90 μs; 135 Hz), but patient did not find this as effective for his OCD. Ultimately, he remains on monopolar settings without hypomania and manages building irritability by switching to bipolar settings (usually once or twice a day). He specifically changes to bipolar before driving because he recognizes this is a time where he is more prone to irritability, and he also switches to bipolar for sleep.

## Results

### Anecdotal Evidence

#### Patient 1

Despite persistent low BMI of 14, she has remained out of the hospital for 29 months, the longest time period since onset of OCD and anorexia. She is working part time as a research assistant, is active in her church, and, though she wishes for further reduction in symptoms, she notes her quality of life and mood is better than prior to DBS. In addition, she no longer engages in self-injurious behaviors and no longer experiences suicidal ideation.

#### Patient 2

Patient has been volunteering regularly and is happy to find that others enjoy working with him. He has returned to school and learned computer and basic life skills (e.g., doing online banking), which pleases his mother who is worried about his ability to be independent once she dies.

#### Patient 3

He began a healthcare management graduate programming and did very well but decided that was not the career path for him. He is currently thriving in a new graduate program for architectural design.

#### Patient 4

Sustained improvement has only been recent, and he is struggling to determine how to fill his day, given that much of his time was previously occupied by compulsions. He recently started working as a history teacher and is finding this very challenging to do virtually (due to the pandemic and in-person learning restrictions).

#### Patient 5

He is thrilled at his ability to play video games without intrusion from OCD, he took a drawing class, and he has resumed playing in a racquet-ball league. He is considering whether he would like to find a volunteer position vs. apply for a job as a staff accountant.

### Diagnostic Scale Analysis

**Figure 1:**
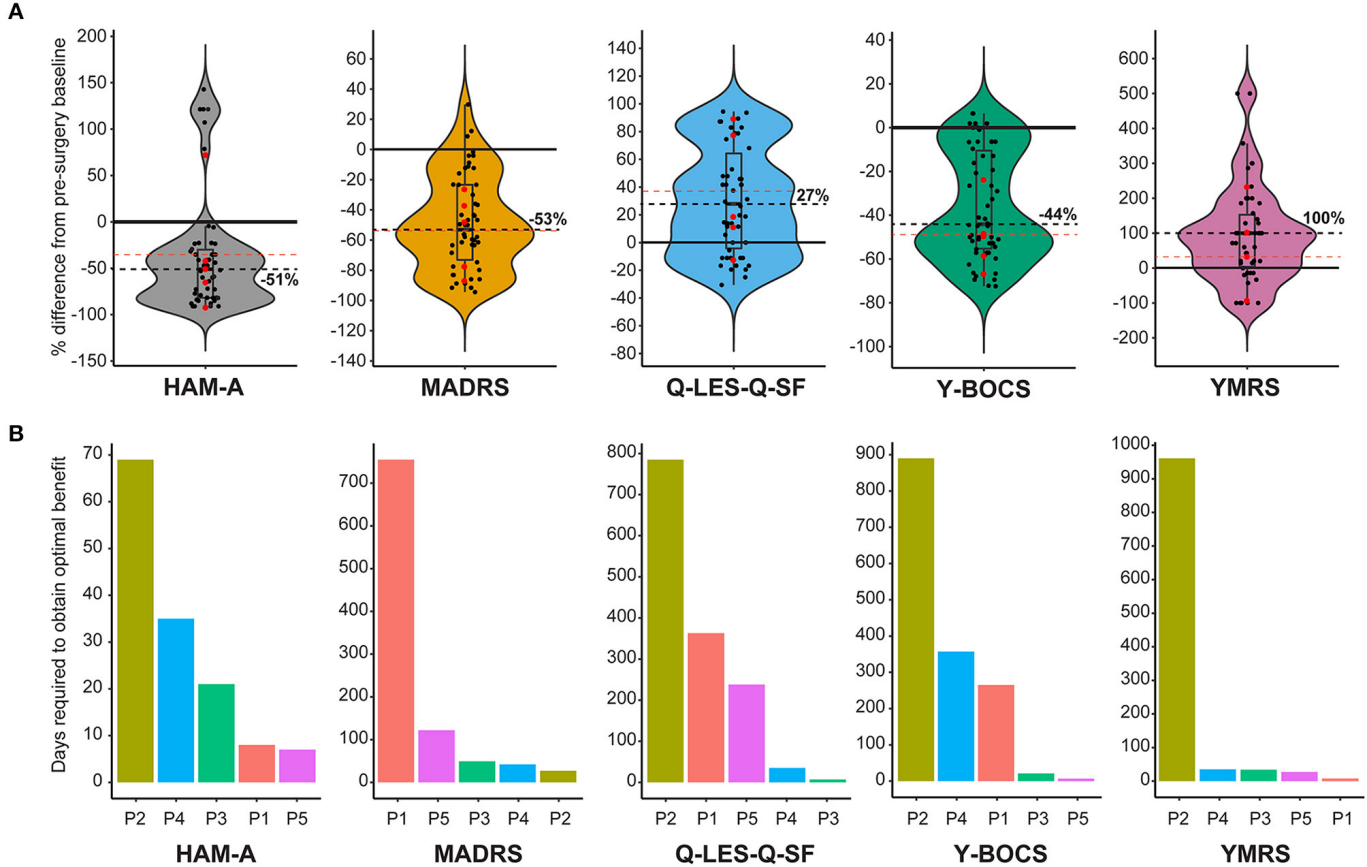
**(A)** Violin plots showing group level representation of the percent change from pre-DBS surgery baseline across the five mood disorder scale metrics used for our cohort: Hamilton Anxiety Rating Scale (HAM-A), Montgomery-Åsberg Depression Rating Scale (MADRS), Quality of Life Enjoyment and Satisfaction Questionnaire (Q-LES-Q-SF), Yale-Brown Obsessive-Compulsive Scale (Y-BOCS), Young Mania Rating Scale (YMRS). Dots represent post-surgery DBS programming sessions. Each scale plot represents all subjects and all post-surgery DBS programming sessions for the first year of follow-up. The solid line demarcates no change (0%) on the y-axis, and the dotted line indicates the median percent change. Red points indicate percent change in scale metrics for each individual patient from the final follow-up and the red dotted line represents the mean for all patients for the final follow up. **(B)** Individual and scale differences in the number of programming sessions necessary to achieve optimal therapeutic stimulation. Each scale plot denotes the number of days required to achieve optimal stimulation for each patient highlighted by a different color.

**Figure 2:**
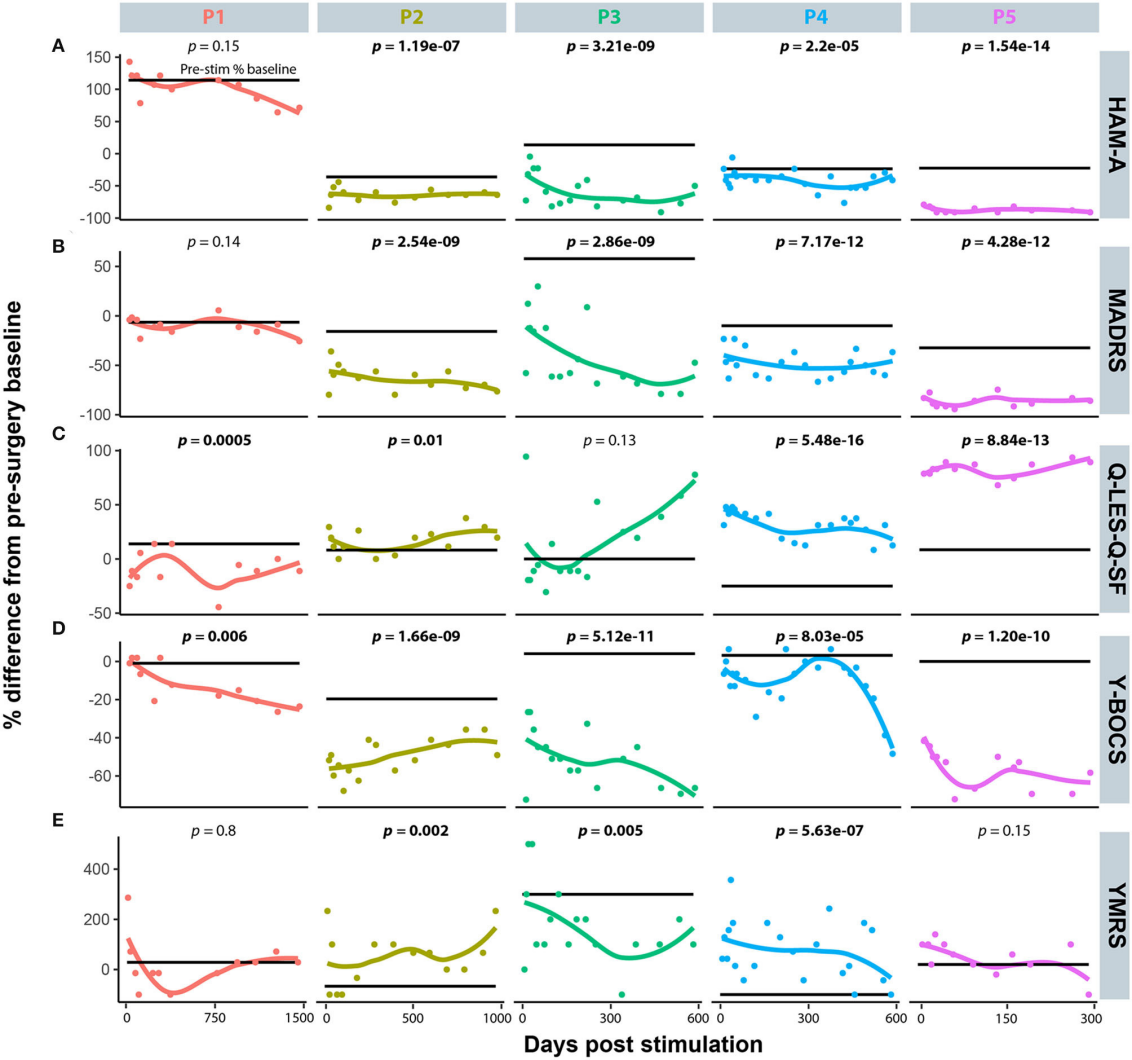
The individual time course representation for patient response to DBS programming modifications across the five mood disorder scale metrics. **(A)** Hamilton Anxiety Rating Scale (HAM-A). **(B)** Montgomery-Åsberg Depression Rating Scale (MADRS). **(C)** Quality of Life Enjoyment and Satisfaction Questionnaire (Q-LES-Q-SF). **(D)** Yale-Brown Obsessive-Compulsive Scale (Y-BOCS). **(E)** Young Mania Rating Scale (YMRS).

Five diagnostic scales were applied to all cases: Y-BOCs, MADRS, and the Q-LES-Q-SF, HAM-A, and YMRS. The median post-stimulation change as a percent of baseline, across all cases for Y-BOCs, was −44% (IQR = 44%) and at final follow-up the mean percent change was −49.1%; MADRS, the median change was −53% (IQR = 49%) and at final follow-up the mean percent change was −54.1%; Q-LES-Q, DBS resulted in a median increase of 27% (IQR = 68%) in perceived quality of life and satisfaction and at final follow-up the mean percent change was 37.6%; YMRS, DBS induced a median increase of 100% and at final follow-up the mean percent change was 32.4%; HAM-A, DBS induced a median reduction of −51% and at final follow-up the mean percent change was −35.0% (Figure 1A). For individual cases, a varying number of post-stimulation parameter adjustments were required to achieve optimal response. Figure 1B shows that most patients showed significant improvement in MADRS, HAM-A and YMRS, by around 100 days post-stimulation, whereas both Q-LES-Q-SF and Y-BOCS required more than 250 days for at least 3 of the cases to achieve peak change from baseline. Variation in response to programming was also evident as a function of time. For Y-BOCS, most cases exhibited a significant improvement in obsessive compulsive behaviors that persisted for the duration of their documented therapy. Figure 2A depicts that 3 of the 5 cases exhibit over 50% improvement in YBOCs; however, there are slight fluctuations between 40 and 60% improvement likely modulated by changes in programming parameters. Despite fluctuations, all 5 cases show a trajectory toward improvement as therapy progresses. Figure 2B highlights the change over time in Q-LES-Q-SF response. For this quality-of-life measurement, 4 out of 5 cases (P2–P5), show marked improvement either at the outset of stimulation (P4 and P5) or as function of changes in programming parameters (P2 and P3). Finally, Figure 2C shows that improvement in MADRS is immediate and invariant over time in 3 out of 5 cases. P1 shows no change and no fluctuations, and P3 shows an immediate improvement that gradually increases over time.

### Comorbid Scale Analysis

**Figure 3:**
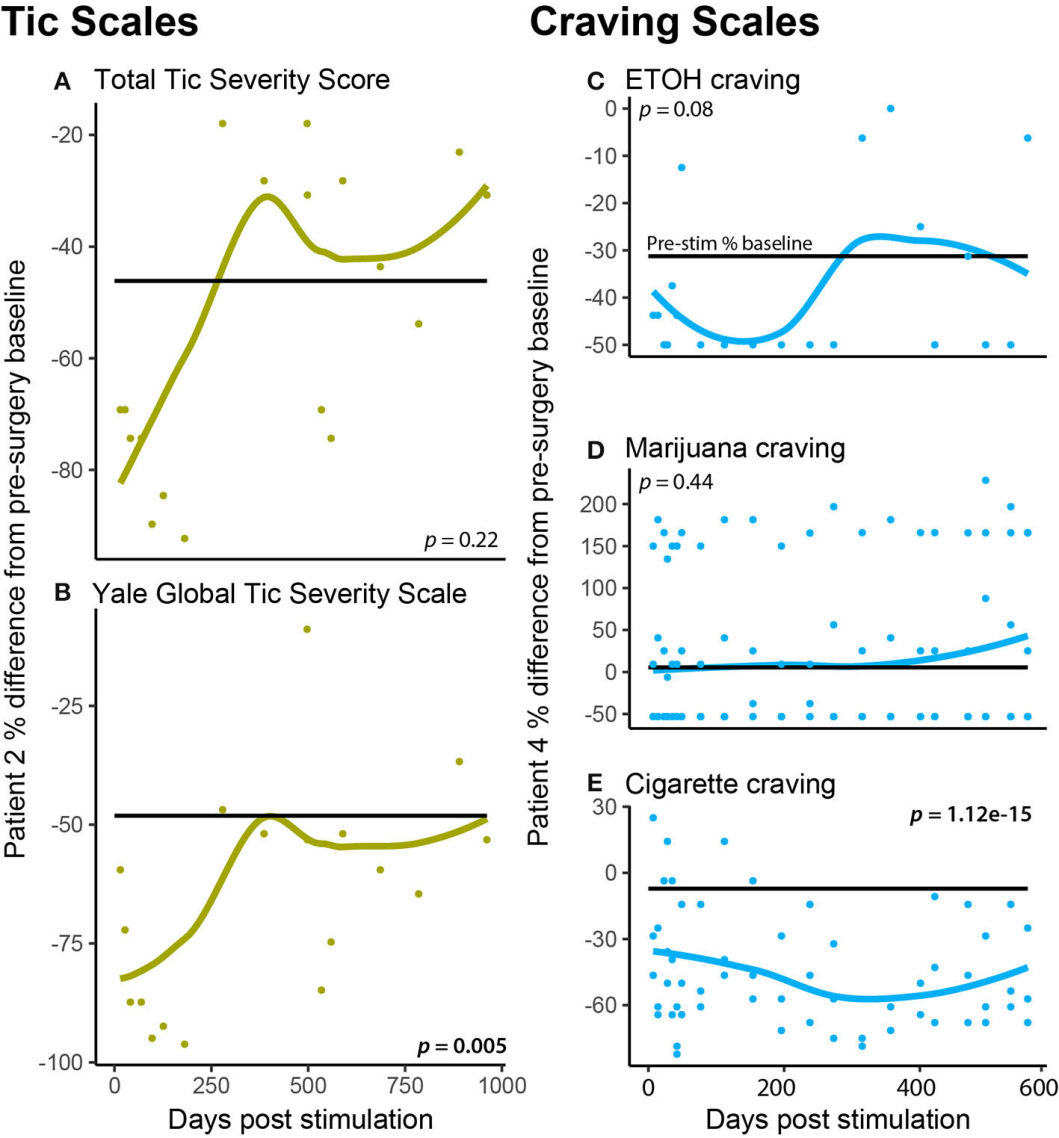
Patient specific comorbidities. **(A,B)** A patient (P2) had a comorbid autism-spectrum disorder, tic disorder, which was measured at each DBS programming session using the **(A)** Total Tic Severity Score and the **(B)** Yale Global Tic Severity Scale **(C–E)**. A patient (P4) had comorbid substance use (nicotine use disorder, daily cannabis use, and at-risk alcohol use), which was measured at each DBS programming session using craving scales for ETOH **(C)**, marijuana **(D)**, and cigarettes **(E)**.

Specific cases in this OCD cohort exhibited comorbid symptoms that in other OCD-DBS reports have responded to DBS. Case 2 had a history of tic disorder manifestations and was assessed pre- and post-surgical using the YGTSS and sub-scale Total Tic Severity Score (TTSS). Figures 3A,B show that TTSS did not significantly change from pre-surgical baseline (*p* = 0.22); however, YGTSS did show a significant reduction that was more marked following initial programming (*p* = 0.005). Case 4 was diagnosed with nicotine use disorder, at risk alcohol use, and daily cannabis use. To assess whether DBS affected the patient's craving for these substances, we measured craving pre- and post-surgery. Figures 3C–E depict Case 4's craving response following DBS. Alcohol craving in Figure 3C shows a marked response during the initial 200 days of stimulation; however, it rebounds back to the pre-stimulation baseline during the latter half of therapy. There is no effect of DBS on marijuana craving overall; however, during many sessions, DBS appears to increase craving. Finally, in Figure 3E, tobacco craving (measured using a cigarette craving rating scale) shows the most lasting response to DBS, with both an immediate and sustained drop in craving by 30%.

### Charge Density Calculated for the Final Follow-up

**Figure 4:**
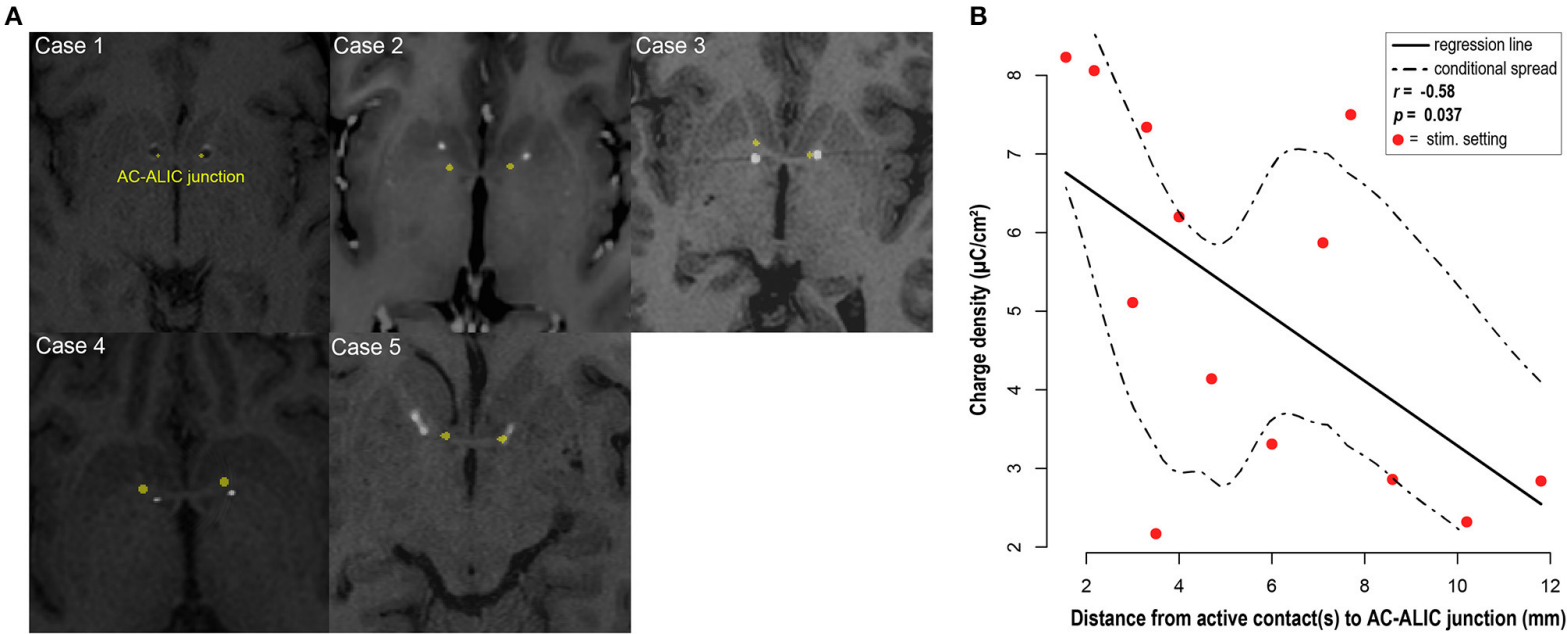
DBS electrode placement. **(A)** For each patient, localization of the bilateral DBS leads is visualized in the axial plane at the level of the anterior commissure (AC) – anterior limb of the internal capsule (ALIC) junction; marked in yellow. Axial slices depict co-registered pre-operative MRI and post-operative CT for cases 2–5 and co-registered pre- and post-operative MRI for case 1. Hyperintense circular artifacts in cases 2–5 represent the DBS lead from the post-operative CT; the hypointense circular artifacts in case 1 represent the DBS lead. Note that the axial images depicting lead location at the junction between AC and ALIC, do not necessarily depict the location of the active contact. **(B)** Visualization and analysis of the association between the distance from the active contact(s) at final follow-up to the AC-ALIC junction and charge density. A significant negative correlation was observed for the relationship between distance between the active contact(s) and the AC-ALIC junction and charge density (*p* = 0.037, *r* = −0.58).

All patients in this cohort experienced improvement in their OCD symptoms as measured by change in Y-BOCS, however the stimulation parameters and selected therapeutic contacts at final follow-up varied across patients. To determine whether an association between anatomical location of therapeutic contacts and tissue activation as measured by charge density, we analyzed the relationship between charge density and distance between the AC-ALIC junction and the mid-point of the active contact(s). We found that the lower the charge density was negatively correlated with the distance between the AC-ALIC junction and the active contact(s); *r* = −0.58, *p* = 0.037 (see Figure 4 and Table 1).

**Table 1:** DBS programming parameters.

**Mean Y- BOCS score pre-surgery**		**6 months**	**Y-BOCS reduction at 6 months**		**Most recent**	**Y-BOCS reduction at last follow-up**	
Patient 1: **35.33**	R PG	(C+, 1–) 6.2 V/150 μs/100 Hz	21% (28) (7 mos)	R	(C+, 0–, 1–) 8 V/120 μs/135 Hz **15.5mA**	1,434 d	26% (27)
	L	(C+, 1–) 7.2/150 μs/100 Hz		L	(C+,0-,1-) 6.7V/150μsec/135Hz **12.8mA**		
Patient 2: **37.33**	R	(0+, 1–) 7 V/60 μs/100 Hz	62% (14)	R	(0+, 1–) 6.5 V/100 μs/100 Hz **6.5mA**	961 d	49% (19)
	L	(0+, 1–) 7 V/60 μs/100 Hz		L	(0+, 1–) 5.0 V/100 μs/100 Hz **5.0mA**		
Patient 3: **32.67** (switches R side between group A and B)	R	(C+, 1–) 3.2 V/120 μs/135 Hz	57% (14)	R A	(C+, 1–) 2.6 V/150 μs/135 Hz **4.9mA**	601 d	66% (11)
				R B	(0+, 1–) 5 V/150 μs/135 Hz **5.9mA**		
	L	(C+, 2–) 4.4 V/120 μs/135 Hz		L	(C+, 2–) 4 V, 150 μs/135 Hz **6.0mA**		
Patient 4: **31**	R	(C+, 1–) 5.5 V/210 μs/135 Hz	19% (25)	R	(C+, 0–, 1–) 4 V/150 μs/135 Hz **8.9mA**	562 d	48% (16)
	L	(C+, 0–) 5.5 V/210 μs/135 Hz		L	(C+, 0–, 1–) 3.5 V/150 μs/135Hz **8.0mA**		
Patient 5: **36** (switches between group Aand group B)	R A	(0–, 3+) 4.5 V/90 μs/135 Hz	69% (11)	R A	(0–, 3+) 4.5 V/90 μs/135Hz **3.8mA**	266 d	58% (15)
	L A	(0–, 3+) 4.2 V/90 μs/135 Hz		L A	(0–, 3+) 4.2 V/90 μs/135 Hz **3.3mA**		
	R B	(C+, 1–) 2.7 V/90 μs/135 Hz		R B	(C+, 1–) 2.7 V/90 μs/135 Hz **3.8mA**		
	L B	(C+, 0–) 2.7 V/90 μs/135 Hz		L B	(C+, 0–) 2.7 V/90 μs/135 Hz **2.9mA**		

*R/L, Right/Left pulsegenerator. Bold values indicate therapeutic current*.

### Adverse Events

Some adverse events were encountered during programming, but these were all temporary. Hypomania was the most encountered adverse effect. Patient 1 had jaw tightening and pulling and tongue tingling. Patient 2 experienced transient hypomania and insomnia after an increase in amplitude, and this resolved without intervention within 1–2 days. Patient 3 had hypomania and sympathomimetic effects including flushing, tachycardia, and hypertension. Patient 4 had dysphoria and irritability at a pulse width of 210. Patient 5 experienced hypomania with irritability and aggression.

## Discussion

While the efficacy of DBS for OCD has been well-established, there are few reports of success in patients with comorbidities, despite the reality that most patients have comorbid psychiatric diagnoses in addition to OCD (25). Here we report the results of VC/VS DBS in five patients whose comorbidities include substance use disorders, MDD, autism spectrum disorder, psychosis, anorexia nervosa, and tic disorder. A recent study of quality of life QOL in OCD demonstrated that QOL in OCD is often as dependent on the comorbid psychiatric disease as the OCD itself (26). This underscores the fact that “success” from DBS in these patients is heavily dependent on their comorbidities in addition to their OCD.

### Anorexia

DBS has been performed for anorexia since 2010, when Israel et al. targeted the subgenual cingulate cortex (27). Other targets include the NAc and the bed nucleus of the stria terminalis (28, 29). These studies have been case reports and case series, so high quality recommendations are not available, though 1-year results of an open label trial at University of Toronto for subcallosal cingulate stimulation demonstrate improvement in body mass index and affective symptoms (30). Comorbid anorexia and OCD have previously been treated by both anterior capsulotomy or VC/VS DBS with improvement in both disorders (31, 32).

### Autism

Case reports have demonstrated improvement in both YBOCS and autism spectrum disorder (ASD) in a patient with co-morbid OCD and autism who underwent stimulation of the NAc (33) and of another patient whose NAc was targeted for isolated self-injurious behavior (SIB) in the setting of ASD (34). Other targets reported for SIB in ASD include the basolateral amygdala (35), globus pallidus interna (GPi), and GPi together with ALIC (36), with improvement in the first two cases but only temporary improvement in the third.

### Tourette's

DBS has been studied in Tourette syndrome more robustly. About 200 cases have been reported in the literature, and five randomized controlled trials comprising a total of 43 patients have been reported (37). However, the optimal target for Tourette's is still the subject of ongoing debate, as 10 different regions have been suggested in the aforementioned studies, including the GPi (anteromedial and posteroventrolateral portions), the globus pallidus externus, the NAc, the ALIC, the subthalamic nucleus (STN), and four regions within the centromedial thalamus. A recent multi-institutional retrospective study aimed to determine whether one target is superior to others in resolving tics. The study did not find that one target was superior to others for resolution of tics, but it did find that regions superior, medial, or within the GPi were associated with greater improvement in co-morbid OCD symptoms than those inferior (38).

### Depression

DBS for treatment-resistant depression (TRD) has been the subject of significant controversy. While open label studies of VC/VS demonstrated promising results (39), a previous randomized-controlled RECLAIM study on the subject was halted early due to lack of significant difference between the two arms after 30 patients had been enrolled (40). Interestingly, in a RCT of 25 patients with bilateral VC/VS DBS in the Netherlands, discontinuation of therapy during the crossover phase resulted in reemergence of symptoms (41). Fifty percent response and 30% remission was noted in open-label long-term follow-up of 28 patients receiving subcallosal cingulate stimulation (42). Other targets being investigated for TRD include superolateral branch of medial forebrain bundle and lateral habenula.

### ADHD

No trials of DBS for ADHD have been performed.

### Substance Use Disorder

Most work regarding DBS for addiction remains in translational stages. While NAc is the most commonly considered target, the lateral hypothalamus, medial prefrontal (PFC) cortex, STN, lateral habenula, and insula have also been targeted with promising results in animal models (43).

### Psychosis

The ventral portion of the CA1 region of the hippocampus, PFC, ventral striatum, NAc, substantia nigra, and ventral tegmental area have been posited as potential targets in schizophrenia (44, 45).

DBS of the VC/VS and NAc has been found to be slightly less effective than lesional anterior capsulotomy for OCD in a literature review, though the groups compared were not exactly analogous since those treated with DBS were more likely to have more severe disease and for a longer time, but it seems that the modulatory nature of DBS makes it more socially acceptable in the fraught world of psychiatric surgery than creation of a permanent lesion (10). Side-effect profiles were similar in both groups. However, as our ability to identify connectivity pathways improves, so too may we be able to predict which patients are the most likely to respond positively to DBS: recent hypotheses focus on the medial and lateral PFC and frontothalamic radiation (46). Another recent study has demonstrated that PFC-related cognitive control, including theta oscillations, improves after DBS of VC/VS (47).

In previous meta-analysis, obsessions and compulsions with sexual and/or religious content are more likely to respond to DBS than other types of compulsions (12). It has also been posited that CBT post-operatively may augment the efficacy of DBS; however, this has only been demonstrated preliminarily in an open-phase trial (48).

### Challenges of Programming

This case series highlights many challenges that psychiatrists may face during programming. Patients may feel markedly improved with initial programming (particularly with regards to mood), and, unfortunately, this degree of improvement does not always persist. This may lead patients to feel disappointed and “chase” the good feeling. To limit the chance of this, the primary programmer has learned to increase amplitude very gradually and only to test higher amplitude during initial programming if response is not evident at lower amplitudes. Programming is more difficult in patients who do not have a good awareness of their internal states or emotions. The programmer may need to rely on more objective observations: for example, increased talkativeness, changes in affect, and degree of indecision. It is useful to have friends and/or family members in the room during programming as this allows for more natural conversation and observation of interactions. It can be helpful to discuss a topic of interest to the patient in order to observe his or her level of interest, engagement, and spontaneity. Additionally, patients with OCD often have trouble making decisions, and providing self-ratings during programming is no exception. Some patients have tended to rate their symptoms (on a scale of 0–10) in increments of 0.25, not wanting to mistakenly over-report changes. Again, the programmer can rely on more objective observations in these cases.

Another challenging aspect of programming is that sometimes patients experience adverse effects at the settings associated with most clinical improvement, such as feeling physically anxious, being more irritable, experiencing insomnia, or having sympathomimetic effects. As described above, one patient manages increased irritability by changing settings depending on context. Another patient changes settings for sleep. Psychiatrists may need to add medication for insomnia. Adjustments to pulse width and frequency may mitigate adverse effects in some patients. The programmer must allow adequate time between changes in settings so that effects from a previous setting do not carry over to the next setting. Sometimes a change is very clear, and the programmer can make changes within 30–60 s. Other times, it is less clear, and the programmer may have the patient take a break for 10–20 min or so on one setting then do the same on another to better compare.

As described above, some patients were able to change from one setting to another in order to mitigate side effects or allow for sustained benefit. Other patients were not able to do this effectively. For example, Patient 2 has not been able to turn down DBS (or turn one hemisphere off) at night due to a fear that he will do it incorrectly and “mess up” his DBS. Patient 4 did not tolerate turning amplitude down at night due to feeling significantly more depressed and anxious. One patient (not included in this case series due to having surgery after this manuscript was drafted) developed compulsions related to her DBS, feeling the urge to repeatedly turn DBS on and off in response to obsessions. Some patients may be unable to manage changes appropriately using the patient programmer initially but may be better able to do so farther along the path of DBS programming as their OCD begins to improve.

It is also important to keep in mind that DBS may not mitigate all of a patient's symptoms, but a patient may have a better response to medication with DBS on. As described, patient #4 had residual tic-like compulsions that did not respond to DBS but did respond to addition of an antipsychotic (when a previous trial of an antipsychotic had not been effective). In summary, though an initial algorithm may be followed, DBS programming must be individualized to each patient, and the programmer must be flexible and creative in order to maximize clinical response while minimizing adverse effects.

Finally, while every effort was made to maintain objectivity, we recognize that our un-blinded status and the lack of a control arm are major limitations to our study. Nonetheless, we feel our case series data are worth sharing given the dearth of co-morbid psychiatric disease in more formal studies of DBS for OCD.

### Conclusions

DBS has proven to be an efficacious treatment with an acceptable side effect profile for treatment of refractory OCD. Here, we have reported our institutional experience with five patients, all with significant co-morbidities. Furthermore, we report programming parameters, which have been seldom discussed in the literature. While these represent only retrospective data, they aid in our corpus of knowledge regarding bilateral VC/VS DBS for OCD and underscore the need for more high-quality Level I evidence regarding surgical management of OCD.

## Data Availability Statement

The raw data supporting the conclusions of this article will be made available by the authors, without undue reservation.

## Ethics Statement

The studies involving human participants were reviewed and approved by Colorado Multiple Institutional Review Board. The patients/participants provided their written informed consent to participate in this study. Written informed consent was obtained from the individual(s) for the publication of any potentially identifiable images or data included in this article.

## Author Contributions

LK, AA, and RD conceived of the manuscript. JT and BS performed statistical analyses. LK, AA, and RD provided direct care for the patients. JT created the figures. JT, AA, LK, RD, BS, and HW contributed directly to the writing and editing of the manuscript. All authors contributed to the article and approved the submitted version.

## Conflict of Interest

LK has received grant funding from Medtronic in the past. RD provides *ad hoc* paid consulting for Medtronic. RD participated in a BrainsWay TMS for OCD Advisory Board in 2019. The remaining authors declare that the research was conducted in the absence of any commercial or financial relationships that could be construed as a potential conflict of interest.
